# Contributions of UDP-Glucuronosyltransferases to Human Hepatic and Intestinal Metabolism of Ticagrelor and Inhibition of UGTs and Cytochrome P450 Enzymes by Ticagrelor and its Glucuronidated Metabolite

**DOI:** 10.3389/fphar.2021.761814

**Published:** 2021-10-14

**Authors:** Shuaibing Liu, Lei Hou, Cai Li, Yibo Zhao, Xia Yao, Xiaojian Zhang, Xin Tian

**Affiliations:** Department of Pharmacy, The First Affiliated Hospital of Zhengzhou University, Zhengzhou, China

**Keywords:** ticagrelor, ticagrelor-O-glucuronide, UDP-glucuronosyltransferases, human liver microsomes, human intestinal microsomes, inhibition, cytochrome P450 enzymes

## Abstract

Ticagrelor is the first reversibly binding, direct-acting, oral P2Y_12_ receptor inhibitor. The contribution of UDP-glucuronosyltransferases (UGTs) enzymes to the metabolism of ticagrelor to its glucuronide conjugation, ticagrelor-O-glucuronide, in human liver microsomes (HLM) and human intestinal microsomes (HIM), was well characterized in the current study. The inhibition potential of human major UGTs by ticagrelor and ticagrelor-O-glucuronide was explored. The inhibitory effects of ticagrelor-O-glucuronide on cytochrome P450s (CYPs) enzymes were investigated as well. Ticagrelor glucuronidation exhibits substrate inhibition kinetics in both HLM and HIM with apparent K_m_ values of 5.65 and 2.52 μM, V_max_ values of 8.03 and 0.90 pmol min^−1^·mg protein^−1^, K_si_ values of 1,343.0 and 292.9 respectively. The *in vitro* intrinsic clearances (*V*_max_/*K*_m_) for ticagrelor glucuronidation by HLM and HIM were 1.42 and 0.36 μl min^−1^·mg protein^−1^, respectively. Study with recombinant human UGTs suggested that multiple UGT isoforms including UGT1A9, UGT1A7, UGT1A3, UGT1A4, UGT1A1, UGT2B7 and UGT1A8 are involved in the conversion of ticagrelor to ticagrelor-O-glucuronide with UGT1A9 showing highest catalytic activity. The results were further supported by the inhibition studies on ticagrelor glucuronidation with typical UGT inhibitors in pooled HLM and HIM. Little or no inhibition of UGT1A1, UGT1A3, UGT1A4, UGT1A6, UGT1A9 and UGT2B7 by ticagrelor and ticagrelor-O-glucuronide was noted. Ticagrelor-O-glucuronide also exhibited limited inhibitory effects toward CYP2C8, CYP2D6 and CYP3A4. In contrast, ticagrelor-O-glucuronide weakly inhibited CYP2B6, CYP2C9 and CYP2C19 activity with apparent IC_50_ values of 45.0, 20.0 and 18.8 μM, respectively. The potential of ticagrelor-O-glucuronide to cause drug-drug interactions warrant further study.

## Introduction

**FIGURE1:**
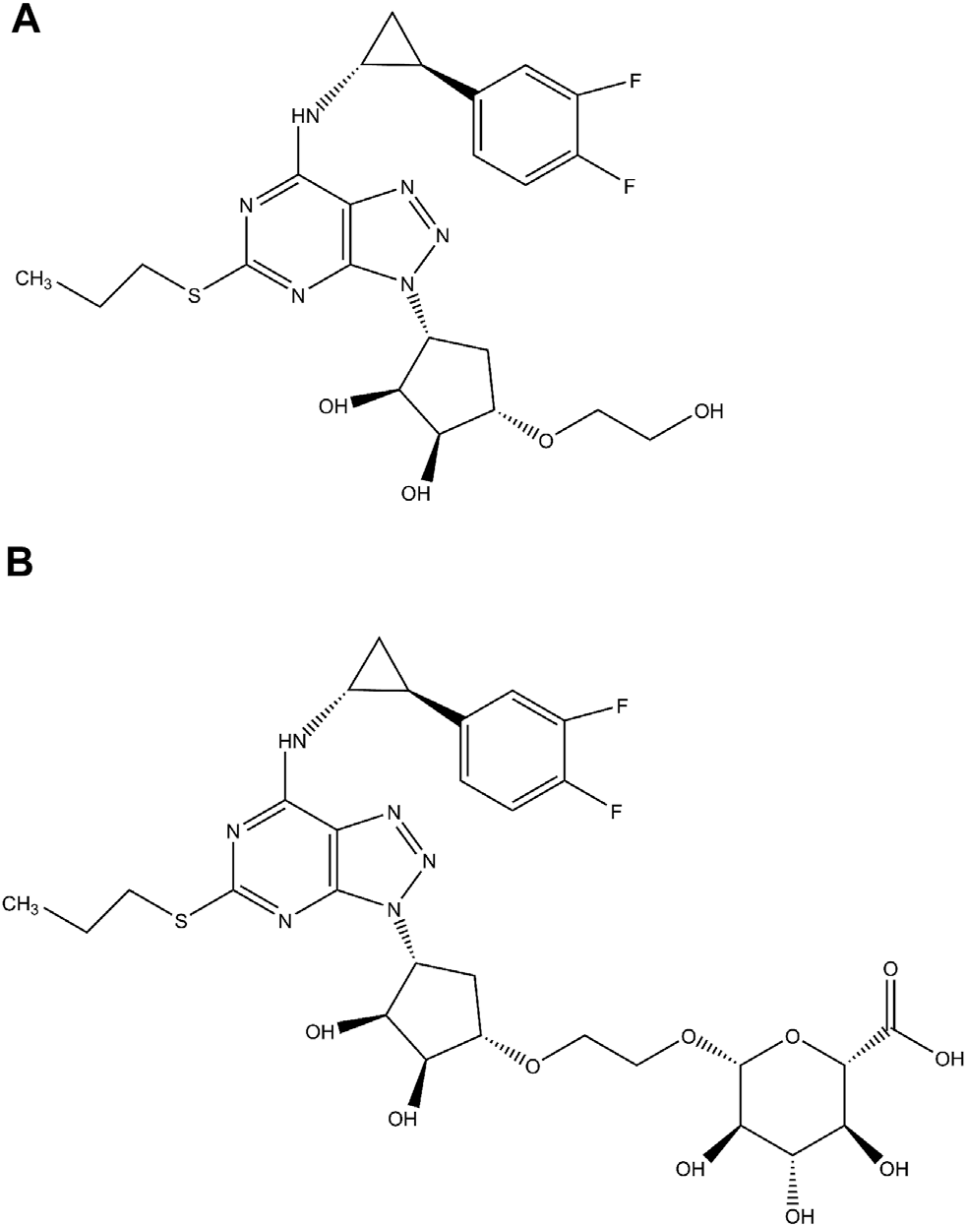
The chemical structures of ticagrelor **(A)** and ticagrelor-O-glucuronide **(B)**.

P2Y_12_ receptor inhibitors remain the cornerstone of the antiplatelet therapy in patients with acute coronary syndromes. Ticagrelor (Figure 1A) is the first reversibly binding, direct-acting, oral P2Y_12_ receptor inhibitor that exhibits more consistent and rapid platelet inhibition than clopidogrel (Gurbel et al., 2009; Husted and van Giezen, 2009). Although ticagrelor does not need metabolic activation to exert its antiplatelet effects, it is still extensively metabolized to the major active metabolite, AR-C124910XX, and to a lesser extent, the inactive metabolite, AR-C133913XX, after ingestion (Teng et al., 2010). Phase I metabolic enzymes, cytochrome P450 (CYP) 3A4 and 3A5 are the major enzymes responsible for the formation of its active and inactive metabolites (Zhou et al., 2011). Although the contribution of phase I metabolic enzymes, CYP3A4 and CYP3A5, to the metabolism of ticagrelor has been well characterized, the role of phase II metabolic enzymes, UDP-Glucuronosyltransferases (UGTs), play in the disposition of ticagrelor remains to be elucidated.

The clearance and detoxification of numerous endogenous (e.g., bilirubin, bile acids, estrogen, etc.) and exogenous compounds (e.g., drugs, nondrug xenobiotics, etc.) are largely attributed to the glucuronidation catalyzed by UGTs isoforms (Miners and Mackenzie, 1991; Radominska-Pandya et al., 1999; Tukey and Strassburg, 2000). Currently, 19 functional human UGTs have been identified. They are classified into subfamilies, 1A, 2A, and 2B according to gene structure and sequence homology (Mackenzie et al., 2005). The UGTs could be detected in various tissues, i.e., liver, gastrointestinal tract, kidneys, and others (Turgeon et al., 2001; Gregory et al., 2004; Mackenzie et al., 2005). Liver and intestine are the major organs mediating the first-pass metabolism of drugs that are substrates of UGTs. Distinct, but overlapping substrate and inhibitor selectivities have been documented for the individual UGT enzymes (Tukey and Strassburg, 2000; Kiang et al., 2005).

The current study was conducted to investigate the contribution of each UGT isoforms to the glucuronidation of ticagrelor in human liver and intestine using human liver microsomes (HLM), human intestinal microsomes (HIM), and recombinant UGTs. The inhibition potential of human major UGT by ticagrelor and ticagrelor-O-glucuronide (ticagrelor-O-Glu, Figure 1B) was explored. The inhibitory effects of ticagrelor-O-Glu on CYP enzymes were explored as well.

## Materials and Methods

### Chemicals and Reagents

Reference standard of ticagrelor (purity 95.0%), 7-Ethyl-10-hydroxy-camptothecin (SN-38) glucuronide (purity 97%), chenodeoxycholic acid 24-acyl-β-D-glucuronide (purity 96%), N-Acetyl serotonin β-D-glucuronide (purity 97%), mycophenolic acid β-D-glucuronide (purity 96%), 3′-azide-3′-deoxythymidine β-D-glucuronide sodium salt (purity 97%), bupropion (purity 98%), hydroxy bupropion (purity >98%), amodiaquine (purity >95%), N-Desethyl amodiaquine hydrochloride (purity >95%), 4′-hydroxylation diclofenac (purity >95%), S-mephenytoin, S**-**4**-**hydroxy mephenytoin (purity >98%), dextromethorphan (purity >95%), dextrorphan tartrate salt (purity >98%), and furafylline (purity >95%, internal standard) were purchased from Toronto Research Chemicals (Toronto, ON, Canada). Reference standard of ticagrelor O-Glu (purity 96.5%) and trifluoperazine N-glucuronide (purity>96%) were purchased from TLC Pharmachem (Concord, ON, Canada). Reference standards of atazanavir (purity 98%), celastrol (purity 98%), SN-38 (purity 99%), chenodeoxycholic acid (purity 98%), trifluoperazine (purity 99%), mefenamic acid (purity 98%), mycophenolic acid (purity >95%), 3′-azide-3′ deoxythymidine (purity 98%), hecogenin (purity 80%), alamethicin (purity >98%) and uridine 5′-diphosphoglucuronic acid trisodium salt (UDPGA, purity >95%) were purchased from J&K Scientific (Beijing, China). N-Acetyl serotonin (purity 98%) and niflumic acid (purity >98%) was purchased from TCI Development Co., Ltd. (Tokyo, Japan). Testosterone (purity >98%) was purchased from European Pharmacopoeia. 6β-hydroxy testosterone (purity >99%) was purchased from Dr. Ehrenstorfer GmbH (Augsburg, Germany). Diclofenac (purity >98%) was purchased from Aladdin Industrial Corporation (Shanghai, China). Diazepam injection as internal standard was purchased from Tianjin Kingyork Pharm Co., Ltd. (Tianjin, China). Pooled HLMs (pool of 150 subjects, mixed gender) and pooled HIMs (pool of 15 subjects, mixed gender) were purchased from XenoTech LLC (Kansas, KS, United States). Recombinant human UGTs Supersomes, such as UGT1A1, UGT1A3, UGT1A4, UGT1A6, UGT1A7, UGT1A8, UGT1A9, UGT1A10, UGT2B4, UGT2B7, and UGT2B17 expressed by baculovirus-infected insect cells were purchased from BD Gentest (Woburn, MA, United States). Formic acid was purchased from Sigma-Aldrich (St Louis, MO, United States). Methanol and acetonitrile of mass spectrometry grade were purchased from Thermo Fisher Scientific (Pittsburg, PA, United States). Purified water was prepared using a Milli-Q water purification system from Millipore Corporation (Billerica, MA, United States). All other chemicals were of the highest purity commercially available.

### Assay of Ticagrelor Glucuronidation

Ticagrelor glucuronidation was investigated in an *in vitro* incubation system by using HLM, HIM, and recombinant human UGTs. The incubation system consisted of enzyme sources (HLM, HIM, or human recombinant UGTs), various concentrations of ticagrelor (dissolved in 50% methanol, 1% v/v, 1, 2, 5, 10, 20, 50, 100, 200, 500 μM of final concentration), alamethicin (50 μg/mg protein), 5 mM MgCl_2_, 100 mM tris-HCL buffer (pH 7.4), and 5 mM UDPGA in a total volume of 100 μl. Alamethicin is a pore-forming polypeptide used to activate UGTs in HLM and HIM (Fisher et al., 2000). Preliminary studies were conducted to determine the optimal protein concentrations and incubation times. Linear conditions of ticagrelor-O-Glu formation from ticagrelor, i.e., HLM, 0.2 mg of protein/ml and 60 min, HIM, 0.2 mg of protein/ml and 60 min, and recombinant human UGTs, 0.2 mg of protein/ml and 60 min were selected. The incubation mixture without UDPGA was placed on ice for 15 min to activate UGT by alamethicin. After pre-incubation for 5 min at 37°C, the reactions were started by the addition of UDPGA. After another 60 min of incubation at 37°C, the reaction was terminated by adding 100 μl of acetonitrile containing internal standard (diazepam). The mixture vortexed for 2 min and then centrifuged at 14,000 rpm for 10 min at 4°C. The resulting supernatant was injected into a liquid chromatography tandem mass spectrometry (LC-MS/MS, Applied Biosystems/MDS Sciex, Foster City, CA) system for the detection of ticagrelor-O-Glu.

### Effects of Typical UGT Inhibitors on Ticagrelor Glucuronidation by Pooled HLM and HIM

The final concentrations of UGT inhibitors for each UGT isoform were set in accordance with previous publications as follows: atazanavir, 1 μM, UGT1A1; celastrol, 5 μM, UGT1A3; hecogenin, 1 μM, UGT1A4; niflumic acid, 1 μM, UGT1A9; and mefenamic acid, 20 μM, UGT2B7 (Lee et al., 2019). The assays were performed as described above, with 10 μM ticagrelor. The percentage of remaining glucuronidation activity was expressed by the following equation:

Remaining activity(%)=Concentration of ticagrelor-O-Glu with inhibitor Concentration of ticagrelor-O-Glu without inhibitor×100.

### UGT Inhibition by Ticagrelor and Ticagrelor-O-Glu

To assess drug-drug interactions (DDIs) potential caused by ticagrelor and ticagrelor-O-Glu through the inhibition of the major human UGTs enzymes, selective UGT probe substrates were co-incubated with ticagrelor or ticagrelor-O-Glu in HLM. The formation of metabolites, SN-38 glucuronide, chenodeoxycholic acid 24-acyl-β-D-glucuronide, trifluoperazine N-glucuronide, N-Acetyl serotonin glucuronide, mycophenolic acid glucuronide and 3′-azide-3′-deoxythymidine β-D-glucuronide, which could reflect the enzyme activities of UGT1A1, UGT1A3, UGT1A4, UGT1A6, UGT1A9 and UGT2B7, respectively, were quantified using LC-MS/MS. Three different concentrations of ticagrelor or ticagrelor-O-Glu (1, 10 and 100 μM) were added to the incubation system in the presence of selective UGT probe substrates (SN-38, chenodeoxycholic acid, trifluoperazine, N-Acetyl serotonin, mycophenolic acid and 3′-azide-3′-deoxythymidine), respectively. The assays were performed as described above. The control was treated with a vehicle solvent only. A triple determination was performed.

### CYPs Inhibition by Ticagrelor-O-Glu

The potential inhibitory effects of ticagrelor-O-Glu on the activities of major CYPs, i.e., CYP2B6, CYP2C8, CYP2C9, CYP2C19, CYP2D6, and CYP3A4, were evaluated by co-incubation ticagrelor-O-Glu with selective P450 probe substrates in HLM. Each incubation mixture containing 0.2 mg/ml HLM, ticagrelor-O-Glu and respective substrate (the final substrate concentrations are close to or less than the K_m_ values: 150 μM bupropion, 1.26 μM amodiaquine, 10 μM diclofenac, 20 μM mephenytoin, 20 μM dextromethorphan and 80 μM testosterone (in house data)) in potassium phosphate buffer (100 mM, pH 7.4) was pre-incubated at 37°C for 5 min. Then, NADPH (10 mM) was added to initiate the reaction. The final volume of the incubation mixture was 100 μl. After incubation at 37°C for specific time points, the reaction was terminated by the addition of 200 μl of ice-cold methanol solution with internal standards (diazepam and furafylline). Preliminary screening experiments with selected concentrations of ticagrelor-O-Glu (0, 1, 10, or 100 μM of the final concentration) were carried out to determine the inhibition potency. For those that produced approximately 50% inhibition of CYPs enzyme activities at a ticagrelor-O-Glu concentration between 1 and 100 μM, IC_50_ was further determined with various concentrations of ticagrelor-O-Glu (0, 1, 5, 10, 20, 50, and 100 μM). All the experiments were performed in triplicates. The supernatant was obtained after centrifugation at 14,000 rpm for 10 min and injected into LC-MS/MS for the analysis of the respective metabolites. The formation of respective metabolites was determined to reflect P450 isoform activities. The metabolites quantified for CYP2B6, CYP2C8, CYP2C9, CYP2C19, CYP2D6, and CYP3A4 were hydroxy bupropion, N-Desethyl amodiaquine, 4′-hydroxylation diclofenac, S-4-hydroxy mephenytoin, dextrorphan and 6β-hydroxy testosterone, respectively.

### LC-MS/MS Analysis

The measurement of ticagrelor-O-Glu and metabolites of UGTs and CYPs substrates was conducted using an LC-MS/MS system composed of Exion LC liquid chromatograph and QTRAP 4500 triple quadrupole mass spectrometer using multiple reaction monitoring (MRM) in both the positive and negative electrospray ionization mode (AB Sciex, Foster City, CA). The chromatographic separation was performed on a Kinetex C18 column (2.1 × 100 mm, 2.6 μm, Phenomenex) with the mobile phase consisting of purified water containing 1% formic acid (A) and methanol (B) with linear gradient elution. The linear gradient program was as follows: 0–1 min, 20% B; 1–5 min, 20–80% B; 5–7 min, 80% B; 7–8 min, 80–20% B; 8–9.5 min, 20% B. The autosampler temperature was maintained at 4°C. A methanol/acetonitrile/isopropanol (1: 1: 1, v/v/v) solution was used as the needle rinse solution. The detailed MRM acquisitions and mass spectrometry voltages for the fifteen analytes are listed in Supplementary Table S1. Other operating parameters were set as follows: curtain gas, collision gas, ion source gas 1 and ion source gas 2 values were set at 35, 7, 40 and 65 psi, respectively. The ion spray voltage was set at 5000 V and −4500 V for the positive and negative mode, respectively. The ion spray temperature was 550°C. The lower limits of quantification of ticagrelor-O-Glu, SN-38 glucuronide, chenodeoxycholic acid 24-acyl-β-D-glucuronide, trifluoperazine N-glucuronide, N-Acetyl serotonin glucuronide, mycophenolic acid glucuronide, 3′-azide-3′-deoxythymidine β-D-glucuronide, hydroxy bupropion, N-Desethyl amodiaquine, 4′-hydroxylation diclofenac, S-4-hydroxy mephenytoin, dextrorphan and 6β-hydroxy testosterone were 0.1, 2.5, 25, 2.5, 25, 25, 25, 2, 1, 10, 10, 1 and 10 ng/ml, respectively. The linearity ranges of the seven analytes were 0.1–100, 2.5–500, 25–5,000, 2.5–500, 25–5,000, 25–5,000, 25–5,000, 2–500, 1–500, 10–2000, 10–5,000, 1–500, and 10–5,000 ng/ml, respectively. The measurement was highly sensitive, selective and reliable. Accuracy and precision met the minimum standards of the bioanalytical method validation guidance for industry released by the U.S. Food and Drug Administration (FDA).

### Enzyme Kinetic Analyses

Kinetic analyses were performed using the mean values of triplicate determinations with either pooled HLM, HIM, or recombinant human UGTs. All data were analyzed by nonlinear regression analysis using the GraphPad Prism 6 (GraphPad Software Inc., CA, United States). Michaelis-Menten kinetics (Eq. 1) or substrate inhibition kinetics (Eq. 2) (Houston and Kenworthy, 2000) were tested to determine the optimal model for describing the data. The optimal model was selected based on the visual inspection of Michaelis-Menten plots:

$$V=V_{\max}\times S/\left(K_{m}+S\right)$$ (1)

$$V=V_{\max}\times S/\left(K_{m}+S+S^{2}/K_{si}\right),$$ (2)

where *V* is the velocity of the reaction, *S* is the substrate concentration, *K*_m_ is the Michaelis-Menten constant, *V*_max_ is the maximum velocity, and *K*_si_ is the substrate inhibition constant. Intrinsic clearance (CL_int)_ was calculated by dividing V_max_ by K_m_.

The percent inhibition relative to the control was calculated to indicate the UGTs or CYPs inhibition. For inhibition exceeding 50% of the control activity, nonlinear regression analysis was used to estimate the IC_50_ values using the GraphPad Prism 6 (GraphPad Software Inc., CA, United States).

### Statistical Analysis

GraphPad Prism 6 (GraphPad Software Inc., CA, United States) was used for statistical analysis. The Kolmogorov–Smirnov test was used to check the normal distribution of continuous data. The statistical comparisons of percentage of control activity (%) between the control and experimental groups were carried out by a two-tailed *t*-test. A *p*-value less than 0.05 was considered statistically significant.

## Results

### Ticagrelor Glucuronidation in Pooled HLM and HIM

**FIGURE 2:**
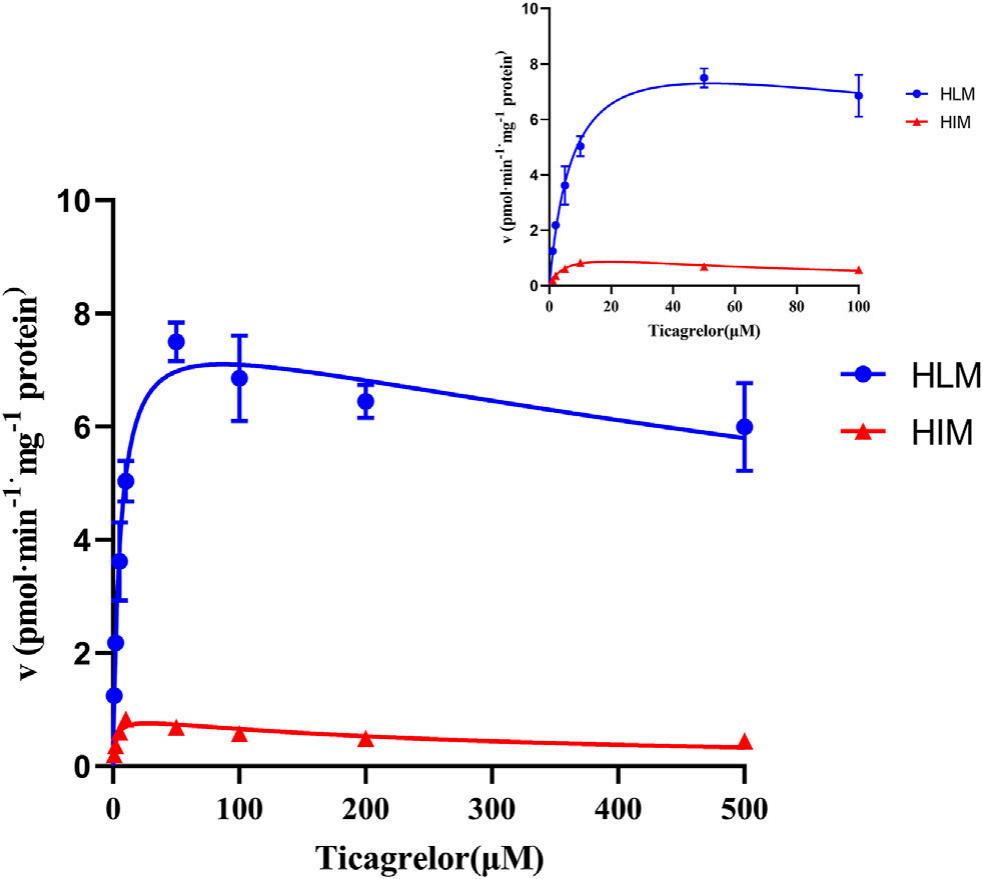
Enzyme kinetics of ticagrelor glucuronidation by HLM and HIM. Each point represents the mean ± S.D. of triple incubations.

The ticagrelor glucuronidation was firstly investigated by selecting various concentrations of ticagrelor utilizing pooled HLM and HIM. The results indicated that the formation of ticagrelor glucuronidation was best described by substrate inhibition kinetics (Figure 2) with apparent K_m_ values of 5.65 and 2.52 μM, V_max_ values of 8.03 and 0.90 pmol min^−1^ mg protein^−1^, K_si_ values of 1,343 and 292.9 respectively. respectively. The *in vitro* intrinsic clearances (*V*_max_/*K*_m_) for ticagrelor glucuronidation by HLM and HIM were 1.42 and 0.36 μl min^−1^·mg protein^−1^, respectively (Table 1).

**TABLE 1:** Kinetic parameters for ticagrelor glucuronidation by HLM, HIM and recombinant human UGT isoforms.

Enzyme sources	K_m_	V_max_	K_si_	CL_int_
	μM	pmol·min^−1^·mg protein^−1^	μM	μl·min^−1^·mg protein^−1^
HLM	5.65	8.03	1,343.0	1.42
HIM	2.52	0.90	292.9	0.36
UGT1A9	1.60	0.90	N.A.	0.56
UGT1A7	1.68	0.12	N.A.	0.07
UGT1A3	19.19	0.78	N.A.	0.04
UGT1A4	10.28	0.36	N.A.	0.04
UGT1A1	7.57	0.20	N.A.	0.03
UGT2B7	14.53	0.23	N.A.	0.02
UGT1A8	6.63	0.09	N.A.	0.01

N.A., not applicable.

### Ticagrelor Glucuronidation by Recombinant Human UGTs

**FIGURE 3:**
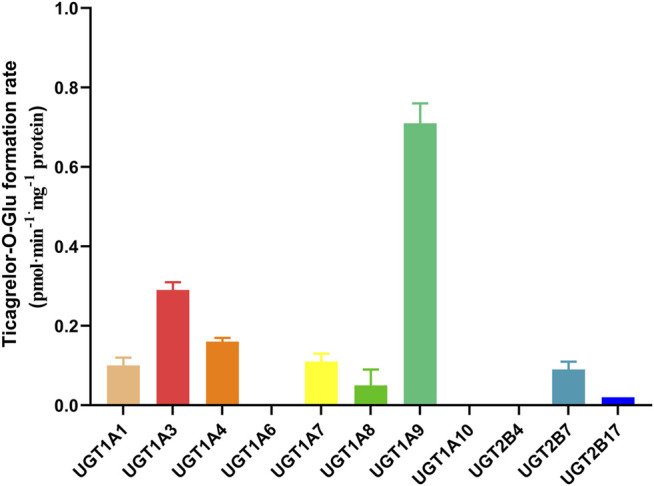
Glucuronidation of 10 µM ticagrelor in human recombinant UGTs. Each column represents the mean ± S.D. of triple incubations.

**FIGURE 4:**
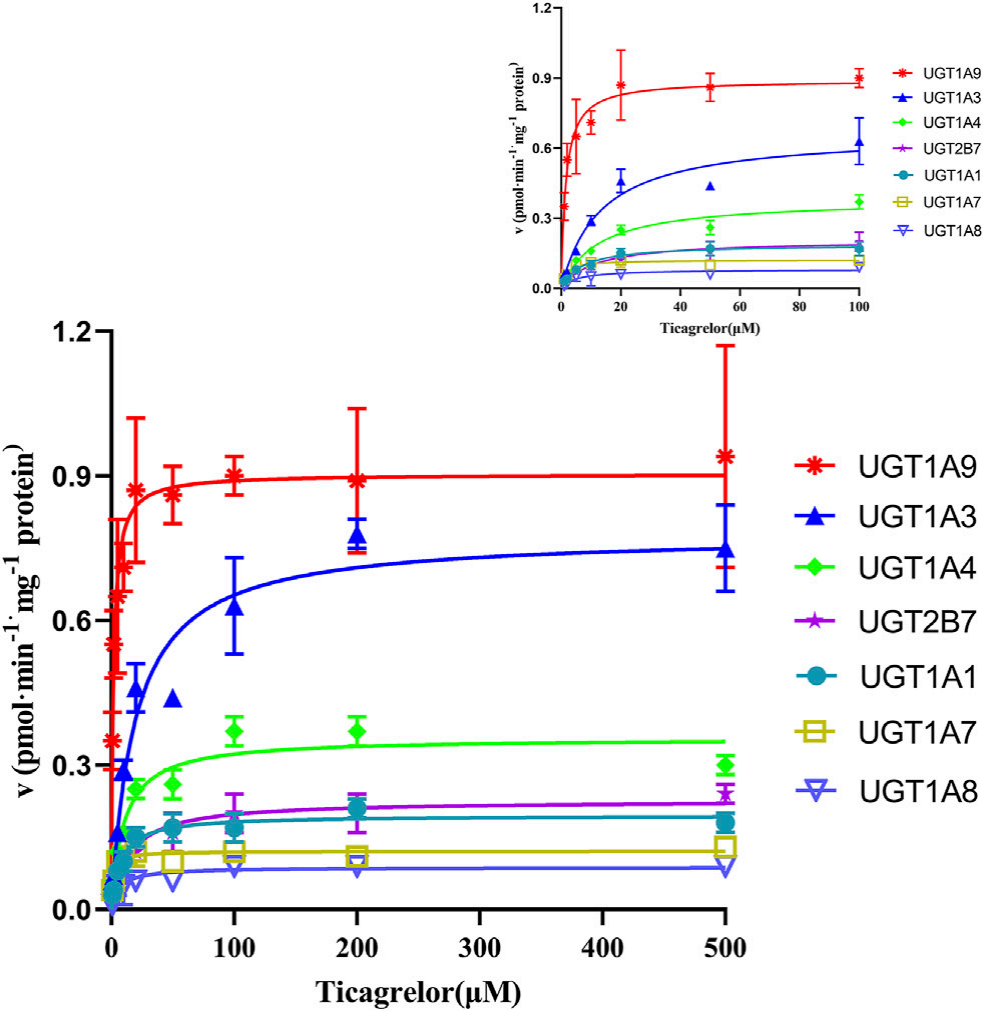
Enzyme kinetics of ticagrelor glucuronidation by human recombinant UGT1A9, UGT1A3, UGT1A4, UGT2B7, UGT1A1, UGT1A7 and UGT1A8. Each point represents the mean ± S.D. of triple incubations.

11 recombinants human UGT isoforms were used for the determination of the contribution of UGTs to the formation of ticagrelor glucuronidation. Single concentration of ticagrelor (10 μM) was used to screen the UGTs isoforms that may participate in the formation of ticagrelor-O-Glu. The results suggested that human UGT1A9, UGT1A3, UGT1A4, UGT2B7, UGT1A1, UGT1A7 and UGT1A8 are all involved in the metabolism of ticagrelor to its glucuronidation metabolite (Figure 3). In contrast, no glucuronidation activities for ticagrelor were noted for other UGT isoforms, i.e., UGT1A6, UGT1A10, UGT2B4, and UGT2B17. Further kinetics study indicated that the kinetics of ticagrelor glucuronidation by recombinant UGT1A9, UGT1A3, UGT1A4, UGT2B7, UGT1A1, UGT1A7 and UGT1A8 fitted Michaelis-Menten kinetics (Figure 4). UGT1A9 exhibited the highest ticagrelor glucuronidation activity with CL_int_ value of 0.56 μl min^−1^·mg protein^−1^, followed by UGT1A7, UGT1A3, UGT1A4, UGT1A1, UGT2B7 and UGT1A8 with the CL_int_ values of 0.07, 0.04, 0.04, 0.03, 0.02 and 0.01 μl min^−1^·mg protein^−1^, respectively. These results clarified the crucial role of UGT1A9, UGT1A7, UGT1A3, UGT1A4, UGT1A1, UGT2B7 and UGT1A8 paly in catalyzing ticagrelor glucuronidation.

### Inhibitory Effects of Typical UGT Inhibitors on Ticagrelor Glucuronidation in Pooled HLM and HIM

**FIGURE 5:**
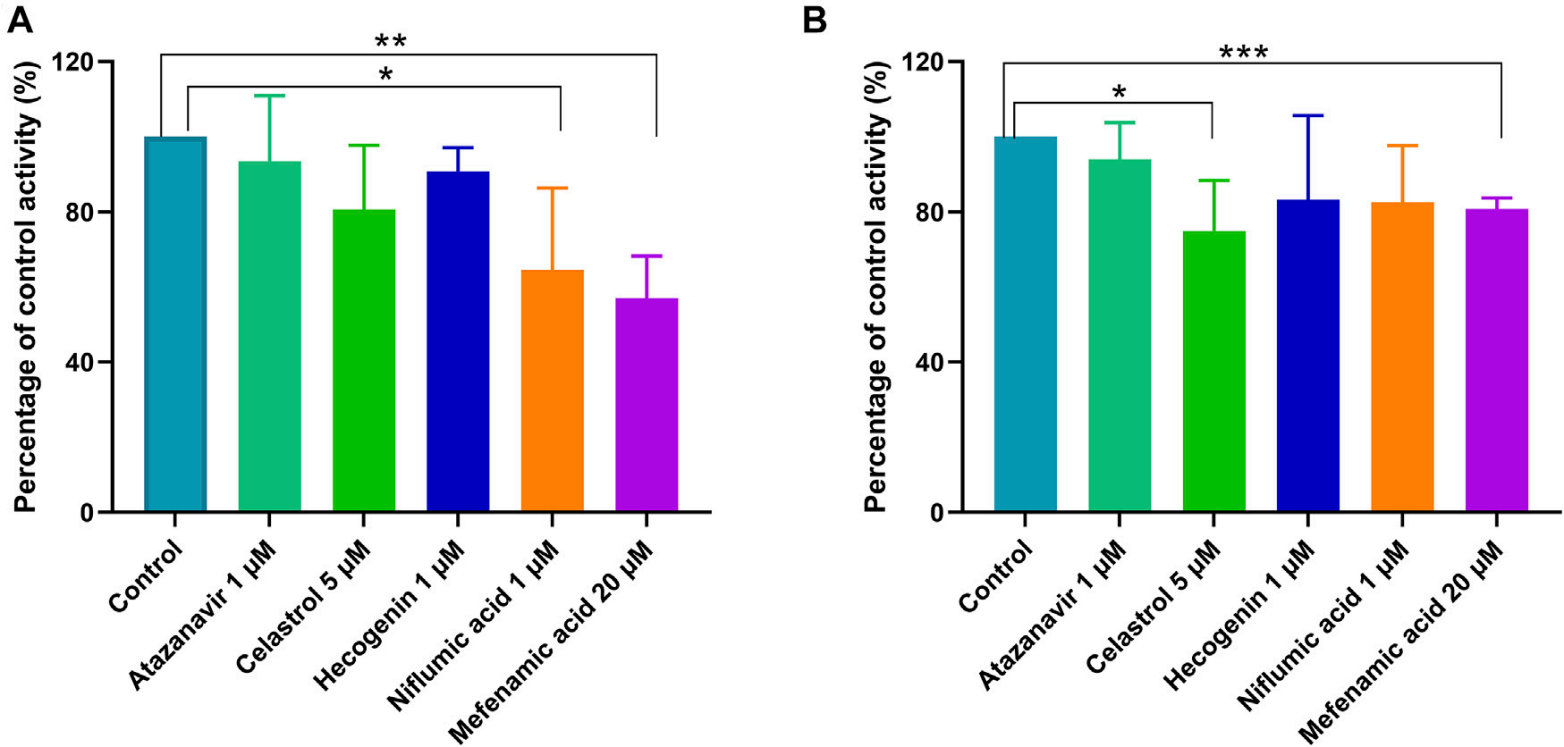
Inhibitory effects of typical UGT inhibitors on ticagrelor glucuronidation in pooled HLM **(A)** and HIM **(B)**. Data represent the mean ± S.D. of triple incubations.

To confirm the results from recombinant enzymes, selective UGTs inhibitors were added to the incubation system individually for the comparison with the formation of ticagrelor glucuronidation in the absence of inhibitors. The results are shown in Figure 5. In HLM and HIM, 35.48 and 17.45%, 19.35 and 25.02%, 9.29 and 16.75%, 6.66 and 6.08%, and 42.95 and 19.24% of ticagrelor-O-Glu formation was inhibited by niflumic acid, the UGT1A9 selective inhibitor, celastrol, the UGT1A3 selective inhibitor, hecogenin, the UGT1A4 inhibitor, atazanavir, the UGT1A1 inhibitor, and mefenamic acid, the UGT2B7 inhibitor, respectively. These results further supported the pivotal role of UGT1A9, UGT1A3, UGT1A4, UGT1A1 and UGT2B7 play in the glucuronidation of ticagrelor in both HLM and HIM.

### UGT Inhibition by Ticagrelor and Ticagrelor-O-Glu

**FIGURE 6:**
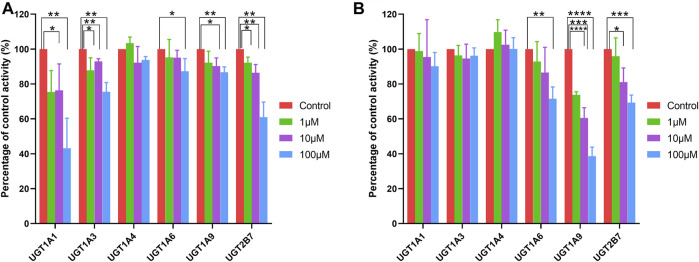
Effects of ticagrelor **(A)** and ticagrelor-O-Glu **(B)** on the activities of human UGT enzymes. SN-38, chenodeoxycholic acid, trifluoperazine, N-Acetyl serotonin, mycophenolic acid, 3′-azide-3′-deoxythymidine were used as the probe substrates for UGT1A1, UGT1A3, UGT1A4, UGT1A6, UGT1A9 and UGT2B7, respectively. Each bar represents the mean ± S.D. of triple incubations. *, *p* < 0.05; **, *p* < 0.01; ***, *p* < 0.001, ****, *p* < 0.0001. Each bar represents the mean ± S.D. of triple incubations.

Selective UGT probes were used to investigate the inhibitory effects of human UGT enzymes by ticagrelor and ticagrelor-O-Glu in HLM. No significant inhibitory effect of ticagrelor on UGT1A4 was noted at ticagrelor concentrations of 1, 10 and 100 μM. In contrast, ticagrelor exhibited a significant inhibitory effect on UGT1A6 only when its concentration was as high as 100 μM. For UGT1A1 and UGT1A9, significant inhibition was observed at ticagrelor concentrations of 10 and 100 μM. All three concentrations of ticagrelor showed significant inhibitory effects on UGT1A3 and UGT2B7. However, for those ticagrelor exerted significant inhibitory effects on, 56.77, 24.50, 12.68, 13.29, and 38.98% inhibition of UGT1A1, UGT1A3, UGT1A6, UGT1A9 and UGT2B7 were achieved at 100 μM of ticagrelor, respectively, indicating the IC_50_ values for these UGT isoforms were close to or greater than 100 μM (Table 2 and Figure 6A). For ticagrelor-O-Glu mediated UGT inhibition, no significant inhibitory effects on UGT1A1, UGT1A3 and UGT1A4 were observed at ticagrelor-O-Glu concentration up to 100 μM. 61.37, 30.69, and 28.50% inhibition of UGT1A9, UGT2B7 and UGT1A6 were observed at 100 μM of ticagrelor-O-Glu, respectively, suggesting the IC_50_ values were close to or greater than 100 μM (Table 2 and Figure 6B).

**TABLE 2:** UGT-selective metabolic pathways inhibition mediated by ticagrelor and ticagrelor-O-glucuronides.

UGTs	Selective pathway	IC_50_ (μM)	
		Ticagrelor	Ticagrelor-O-Glu
UGT1A1	SN-38 glucuronide	≈100.0	>100.0
UGT1A3	Chenodeoxycholic acid 24-acyl-β-D-glucuronide	>100.0	>100.0
UGT1A4	Trifluoperazine N-glucuronide	>100.0	>100.0
UGT1A6	N-Acetyl serotonin glucuronide	>100.0	>100.0
UGT1A9	Mycophenolic acid glucuronide	>100.0	≈100.0
UGT2B7	3′-azide-3′-deoxythymidine β-D-glucuronide	>100.0	>100.0

### CYPs Inhibition by Ticagrelor-O-Glu

**FIGURE 7:**
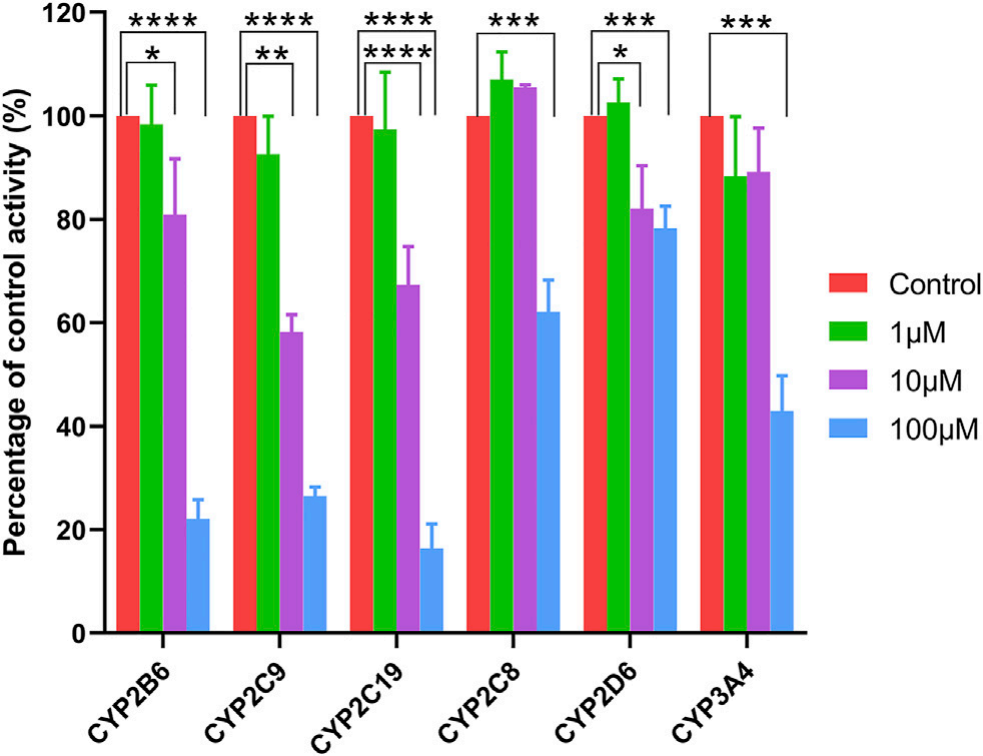
Effects of ticagrelor-O-Glu on the activities of human CYPs enzymes. bupropion, amodiaquine, diclofenac, [S]-mephenytoin, dextromethorphan, and testosterone were used as the probe substrates for CYP2B6, CYP2C8, CYP2C9, CYP2C19, CYP2D6 and CYP3A4, respectively. Each bar represents the mean ± S.D. of triple incubations. *, *p* < 0.05; ***, *p* < 0.001, ****, *p* < 0.0001. Each bar represents the mean ± S.D. of triple incubations.

**FIGURE 8:**
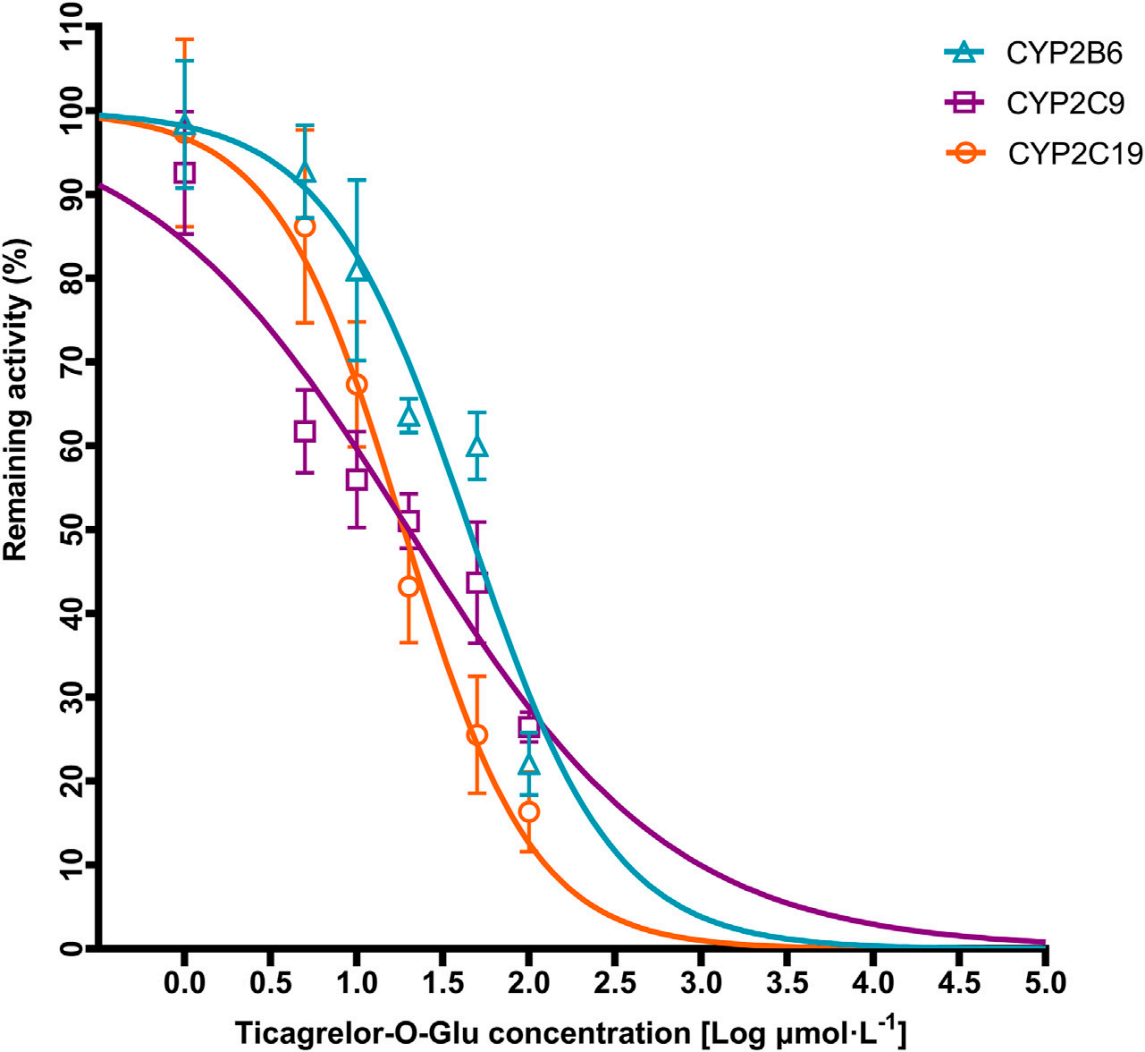
Representative IC_50_ plots demonstrating the inhibitory effect of ticagrelor-O-Glu at concentration of 0–100 µM on the activities of CYP2B6, CYP2C9, and CYP2C19.

Ticagrelor-O-Glu exhibited significant inhibition toward selected CYP isoforms, i.e., CYP2B6, CYP2C8, CYP2C9, CYP2C19, CYP2D6 and CYP3A4. Ticagrelor-O-Glu inhibited CYP2B6 activity ranged from 19.06% at 10 μM to 77.93% at 100 μM. 44.02 and 73.53% as well as 32.67 and 83.63% inhibition of CYP2C9 and CYP2C19 enzyme activities were observed at 10 and 100 μM of ticagrelor-O-Glu, respectively, suggesting the IC_50_ values were between 10 and 100 μM (Table 3 and Figure 7). IC_50_ was further determined considering the obvious inhibitory effects of CYP2B6, CYP2C9 and CYP2C19 by ticagrelor-O-Glu. Weak inhibition of CYP2B6, CYP2C9 and CYP2C19 activities by ticagrelor-O-Glu with apparent IC_50_ values of 45.0, 20.0 and 18.8 μM, respectively was noted (Table 3 and Figure 8). For CYP2C8, CYP2D6 and CYP3A4, significant inhibitory effects of ticagrelor-O-Glu on these isoforms were obtained when the concentration of ticagrelor-O-Glu was as high as 100 μM. However, even for CYP3A4, which ticagrelor-O-Glu exhibited strongest inhibition potency toward, only 57.1% inhibition of enzyme activity was noted, thus IC_50_ values was not further determined for CYP2C8, CYP2D6 and CYP3A4 by ticagrelor-O-Glu.

**TABLE 3:** CYPs-selective metabolic pathways inhibition mediated by ticagrelor-O-Glu.

CYPs	Selective pathway	IC_50_ (μM)
CYP2B6	Bupropion hydroxylase	45.0
CYP2C8	Amodiaquine N-deethylase	>100.0
CYP2C9	Diclofenac 4′-hydroxylase	20.0
CYP2C19	[S]-Mephenytoin 4′-hydroxylase	18.8
CYP2D6	Dextromethorphan O-demethylase	>100.0
CYP3A4	Testosterone 6β-hydroxylase	>100.0

## Discussion

*In vitro* identification of the contribution of UGT enzymes to the hepatic and intestinal metabolism of ticagrelor to its glucuronide conjugation, ticagrelor-O-Glu, was conducted in the current study. Meanwhile, the inhibition potential of human major UGT enzymes by ticagrelor and ticagrelor-O-Glu was explored, the inhibitory effects of ticagrelor-O-Glu on CYPs were investigated as well. The study aimed at providing evidence for evaluation of DDI risk due to the inhibition of UGTs or CYPs metabolic pathway when ticagrelor was co-medicated with UGT or CYPs substrates in clinic.

UGTs play important roles in the metabolism and disposition of numerous endogenous and exogenous compounds (Jarrar and Lee, 2021). However, the contribution of UGTs to human hepatic and intestinal metabolism of ticagrelor remains unknown. Our study is the first to suggest that multiple UGT isoforms including UGT1A9, UGT1A7, UGT1A3, UGT1A4, UGT1A1, UGT2B7, and UGT1A8 are involved in the conversion of ticagrelor to ticagrelor-O-Glu with UGT1A9 showing highest catalytic activity. In contrast, other UGT isoforms, i.e., UGT1A6, UGT1A10, UGT2B4, and UGT2B17 exhibited no glucuronosyltransferase activity toward ticagrelor. UGT1A9 and UGT1A10 are the isoforms that show the highest mRNA expression levels in the liver and intestines, respectively (Ohno and Nakajin, 2009). The results verified the importance of liver UGT1A9 in mediating the formation of ticagrelor-O-Glu. In contrast, UGT1A10, which is specifically expressed in the intestine, made no contribution to the glucuronidation of ticagrelor, implying the ticagrelor glucuronidation in intestine is catalyzed by other UGT isoforms. It is further supported by the inhibition study with UGT inhibitors in HIM.

UGT1A9 is a polymorphic enzyme. Significant variability in the pharmacokinetics of mycophenolic acid exists in subjects carrying different UGT1A9 genotypes (Na Takuathung et al., 2021). Presumably, it may also be a factor responsible for the interindividual variation of ticagrelor. However, the association between a single nucleotide polymorphism (SNP) in UGT2B7 and steady-state area under the curve of AR-C124910XX instead of ticagrelor was identified in the genome-wide association study conducted by Varenhorst et al. (2015). One possible explanation is the altered activity caused by the functional SNP in UGT1A9 or UGT2B7 is compensated by other UGT isoforms since multiple UGT isoforms are involved in the glucuronidation of ticagrelor. Involvement of UGT2B7 enzyme in downstream metabolic step of AR-C124910XX may be another possibility. Further studies toward identifying the role of UGT2B7 in metabolism of AR-C124910XX should be underway.

It is increasingly recognized that the circulating metabolites of drugs play important role in mediating pharmacokinetics DDIs (VandenBrink and Isoherranen, 2010; Yeung et al., 2011). Therefore, it is recommended to investigate the role of major circulating metabolites in DDI study. However, due to the commercial unavailability of most circulating metabolites standards, only limited studies explored the contribution of circulating metabolites to DDIs. Clopidogrel and gemfibrozil are two good examples, revealing their respective glucuronide metabolites, gemfibrozil 1-O-β-glucuronide and clopidogrel acyl-β-D-glucuronide, could both cause serious DDIs as strong time-dependent inhibitors of CYP2C8 (Shitara et al., 2004; Ogilvie et al., 2006; Tornio et al., 2008; Tornio et al., 2014). In addition to metabolites mediated DDIs, the role of the parent drug in DDI is self-evident. Although the inhibitory effects of ticagrelor on CYPs have been thoroughly investigated (Zhou et al., 2011), the inhibition potency of ticagrelor against UGT isoforms is unclear. Based on above facts, the inhibition potential of human major UGT by ticagrelor and ticagrelor-O-Glu was explored. The inhibitory effects of ticagrelor-O-Glu on CYP enzymes were explored as well.

For UGT inhibition, the concentration range of ticagrelor used in this study was 0.52–52.26 μg/ml. In contrast, the steady-state maximal plasma concentrations (*C*_max_) of ticagrelor after a 100 mg dose of b.i.d. in humans was 0.81 μg/ml (Husted et al., 2006). It seems that the concentrations used were greater than the *C*_max_ of ticagrelor. However, regarding metabolism based DDIs, the liver concentration should be used since it is closer to the concentration at the enzyme site. Although the exact concentration in the liver is difficult to predict, it can be estimated from the volume and flow rate of portal blood. In healthy adults, the volume of portal blood space is approximately 450 ml, and portal blood flow rate is ∼600 ml/min (Hofmann et al., 1983). Therefore, approximately 18 L of portal blood will pass through the liver during the first 30 min after an oral dose of 180-mg of ticagrelor. Assuming it is completely absorbed, the concentration that reaches the liver is approximately 10,000 μg/ml or 19 μM. The concentrations used in the current study cover the range of concentrations expected *in vivo*. The results indicated that both ticagrelor and ticagrelor-O-Glu exhibited weak inhibition toward UGT1A6, UGT1A9 and UGT2B7. By contrast, ticagrelor also inhibited UGT1A1 and UGT1A3, which is not noted for ticagrelor-O-Glu. No significant inhibitory effect on UGT1A4 by neither ticagrelor nor ticagrelor-O-Glu at concentrations up to 100 μM. Thus, the DDI risk attributed to the inhibition of these UGT isoforms by ticagrelor and or ticagrelor-O-Glu is low. Regarding the inhibition of CYPs, selected concentrations of ticagrelor-O-Glu were used to do the preliminary screen. Although significant inhibition toward CYP2C8, CYP2D6 and CYP3A4 by ticagrelor-O-Glu was noted when the concentration of ticagrelor-O-Glu is as high as 100 μM, the inhibition potencies were not so strong that IC_50_ values for these isoforms by ticagrelor-O-Glu were not further determined. However, ticagrelor-O-Glu weakly inhibited CYP2B6, CYP2C9 and CYP2C19 activities with apparent IC_50_ values of 45.0, 20.0 and 18.8 μM, respectively.

Metabolites-mediated DDI attributed to the inhibition of cytochrome P450 enzymes by glucuronide metabolites are relatively common in clinical scenarios (Isoherranen et al., 2009). The study provides a new insight into elucidating the role of ticagrelor-O-Glu in ticagrelor-mediated DDIs through the inhibition of CYP2B6, CYP2C9 and CYP2C19. Additional valuable information can be obtained in explaining the underlying mechanism regarding DDIs mediated by ticagrelor. However, to our knowledge, there is only one study that explored the effect of ticagrelor on pharmacokinetic of tolbutamide, a CYP2C9 substrate, in healthy subjects. It is concluded that the possibility of ticagrelor affecting CYP2C9-mediated drug metabolism is extremely low (Teng et al., 2013). No other studies have evaluated the effect of ticagrelor as a perpetrator on CYP2B6 and CYP2C19 substrates. It is important to perform further static and PBPK modeling to predict the DDI risk attribution to the inhibition of CYP2B6 and CYP2C19 by ticagrelor-O-Glu in clinical scenarios. However, due to lack of the exposure data of ticagrelor-O-Glu *in vivo*, it is difficult to evaluate its role in DDI risk in clinic. Even so, it is important to note that although the relatively low concentrations of ticagrelor-O-Glu in comparison to its major active metabolite, AR-C124910XX, the potential of ticagrelor-O-Glu to cause DDIs should also not be ignored. Further study is warranted to reveal the *in vivo* exposure of ticagrelor-O-Glu and investigate the DDI risks caused by ticagrelor-O-Glu as inhibitors of CYP2B6 and CYP2C19.

In summary, the present results indicate that ticagrelor glucuronidation is mainly catalyzed by UGT1A9 in human liver, followed by UGT1A7, UGT1A3, UGT1A4, UGT1A1, UGT2B7, and UGT1A8 in human liver and/or intestine. Little or no inhibition of UGT1A1, UGT1A3, UGT1A4, UGT1A6, UGT1A9 and UGT2B7 by ticagrelor and ticagrelor-O-glucuronide was noted. Ticagrelor-O-Glu also exhibited limited inhibitory effects toward CYP2C8, CYP2D6 and CYP3A4. In contrast, ticagrelor-O-glucuronide weakly inhibited CYP2B6, CYP2C9 and CYP2C19 activity with apparent IC_50_ values of 45.0, 20.0 and 18.8 μM, respectively. When ticagrelor is co-administered with CYP2B6, CYP2C9 and CYP2C19 substrate drugs in clinic, the possibility of clinically significant DDI deserves further study.

## Data Availability

The original contributions presented in the study are included in the article/Supplementary Material, further inquiries can be directed to the corresponding authors.

## Abbreviations

UGTs, UDP-glucuronosyltransferases; HLM, human liver microsomes; HIM, human intestine microsomes; CYPs, cytochrome P450s; Ticagrelor-O-Glu, Ticagrelor-O-glucuronide; SN-38, 7-Ethyl-10-hydroxy-camptothecin; UDPGA, Uridine 5′-diphosphoglucuronic acid trisodium salt; LC-MS/MS, liquid chromatography tandem mass spectrometry; MRM, multiple reaction monitoring; IC50, concentrations of inhibitor causing 50% inhibition of the original enzyme activity; DDI, drug-drug interaction; SNP, single nucleotide polymorphism; C_max_, maximal plasma concentrations.
