# Supplementary material for: Contributions of UDP-Glucuronosyltransferases to Human Hepatic and Intestinal Metabolism of Ticagrelor and Inhibition of UGTs and Cytochrome P450 Enzymes by Ticagrelor and its Glucuronidated Metabolite
Source: Front Pharmacol. 2021 Oct 14;12:761814. doi: 10.3389/fphar.2021.761814 (PMC8552062; doi:10.3389/fphar.2021.761814)
Supplement: Supplementary file 1 [file Table1.DOCX]

Supplementary Table 1. Analytical Conditions and m/z Ratio for Ions of the Compounds

| Enzymes | Analytes | Mode | MRM transition | DP/V | EP/V | CE/V | CXP/V | RT  (min) |
| --- | --- | --- | --- | --- | --- | --- | --- | --- |
| N.A. | Ticagrelor-O-Glu | ESI + | 699.1 → 523.2 | 130 | 10 | 35 | 18 | 5.39 |
| CYP2B6 | Hydroxy bupropion | ESI + | 256.1 → 238.0 | 65 | 8 | 14 | 12 | 3.75 |
| CYP2C8 | N-Desethyl amodiaquine | ESI + | 328.1 → 283.2 | 82 | 10 | 24 | 13 | 1.40 |
| CYP2C9 | 4'-Hydroxydiclofenac | ESI + | 312.1 → 266.1 | 67 | 10 | 17 | 11 | 5.86 |
| CYP2C19 | 4'-Hydroxymephenytoin | ESI + | 235.1 → 150.0 | 86 | 12 | 27 | 6 | 3.68 |
| CYP2D6 | Dextrorphan | ESI + | 258.2 → 199.3 | 83 | 8 | 33 | 9 | 3.41 |
| CYP3A4 | 6β-Hydroxy testosterone | ESI + | 305.0 → 268.9 | 96 | 10 | 20 | 18 | 5.24 |
| UGT1A1 | SN-38-Glicuronide | ESI - | 567.3 → 346.9 | -102 | -6 | -45 | -25 | 4.52 |
| UGT1A3 | Chenodeoxycholic acid-Glucuronide | ESI - | 567.4 → 391.3 | -45 | -4 | -47 | -25 | 6.27 |
| UGT1A4 | Trifluoperazine-Glucuronide | ESI + | 584.2 → 408.0 | 104 | 5 | 34 | 17 | 6.12 |
| UGT1A6 | N-Acetyl Serotion-Glucuronide | ESI + | 395.1 → 219.0 | 60 | 8 | 18 | 9 | 1.40 |
| UGT1A9 | Mycophenolic Acid-Glucuronide | ESI - | 495.2 → 319.0 | -36 | -11 | -26 | -22 | 4.88 |
| UGT2B7 | Zidovudine-Glucuronide | ESI - | 442.3 → 124.8 | -63 | -7 | -29 | -11 | 2.22 |
| IS | Furafylline | ESI - | 258.9 → 177.8 | -76 | -5 | -24 | -13 | 4.44 |
| IS | Diazepam | ESI + | 285.3 → 154.1 | 118 | 11 | 32 | 10 | 6.07 |

N.A. not applicable, IS, internal standard, RT, retention time.
